# Promoting Preconception Care Through Occupational Health Checkups: Development and Pilot Evaluation of a Digital Tool for Japanese Women

**DOI:** 10.7759/cureus.101300

**Published:** 2026-01-11

**Authors:** Tetsuo Morita, Taisuke Morita, Satomi Ooba, Jingo Okawa, Hikohiro Okawa, Toyofumi Hirakawa, Kenichiro Arimura, Kinei Okawa, Eiji Kobayashi, Kazuyoshi Dobashi

**Affiliations:** 1 Obstetrics and Gynecology, Okawa Women's Hospital, Oita, JPN; 2 Obstetrics and Gynecology, Oita University Faculty of Medicine, Yufu, JPN; 3 Neurosurgery and Headache, Ooba Clinic of Neurosurgery and Headache, Oita, JPN; 4 Obstetrics and Gynecology, Faculty of Medicine, Fukuoka University, Fukuoka, JPN; 5 Obstetrics and Gynecology, Senkawa OB/GYN and Breast Clinic, Tokyo, JPN

**Keywords:** digital health, japanese women, occupational health, preconception care, reproductive health, workplace health checkups

## Abstract

Background: Digital approaches to preconception care (PCC) may help increase reproductive health awareness among women of reproductive age. This pilot study evaluated the usability, acceptability, and perceived impact of a digital PCC tool delivered during workplace health checkups in Japan.

Methods: A quick response (QR) code linking to a digital PCC tool was distributed during routine workplace health checkups for women aged 18-40 years. Participants completed a questionnaire assessing perceived usefulness, ease of understanding, relevance to personal health, intention to seek medical consultation, and understanding of PCC. Because not all participants answered every item, denominators varied across outcomes. Age-group differences were examined using chi-square tests.

Results: A total of 277 participants completed at least one questionnaire item and were included in the analysis. Perceived usefulness was high overall (92.8%) with a significant age-group difference (p = 0.001). Ease of understanding also showed a significant difference, with lower ratings in the 35-40 years group (p < 0.001). No significant age-group differences were observed for relevance to personal health (p = 0.795), intention to seek consultation (p = 0.748), or understanding of PCC (p = 0.444). PCC understanding remained high across all age groups (93.8%).

Conclusions: The digital PCC tool was well accepted by women of reproductive age in a workplace setting. Although usefulness and ease of understanding varied by age, other outcomes were consistent across age groups. Further research with larger samples and longitudinal designs is needed to determine whether digital PCC tools influence real-world healthcare engagement.

## Introduction

Workplace health checkups in Japan provide a unique occupational health platform for early identification of health risks and for implementing preventive interventions in working-age populations. Integrating preconception care (PCC) into this system has the potential to promote reproductive health; however, its practical implementation and potential equity implications remain uncertain.

PCC encompasses a range of interventions aimed at optimizing physical, mental, and social well-being before conception [1,2]. When health issues are identified and appropriately managed before pregnancy, related risks can be reduced, ultimately improving outcomes for both mothers and children [3]. Multiple reviews, including Dean et al. (2014), report that uptake of preconception care remains limited globally, particularly among women who are not actively planning pregnancy or who are outside maternal care pathways [4].

In Japan, reproductive health awareness has become increasingly important due to societal trends such as delayed marriage, declining fertility, and postponed childbearing [5]. However, younger women often have limited contact with healthcare services and may be unaware of reproductive health concerns unless information is provided through structured programs [6]. Workplace health checkups, mandated annually under the Industrial Safety and Health Act and covering most employed individuals, offer an opportunity to reach women who may not otherwise seek reproductive health guidance [7]. Nevertheless, PCC has not yet been routinely incorporated into these workplace-based services [8,9].

To address this gap, we developed a digital PCC tool accessible via a quick response (QR) code and designed to provide personalized reproductive health information based on self-reported data. The tool was distributed during workplace health checkups to promote PCC awareness and enhance the perceived relevance of reproductive health issues. This pilot study aimed to evaluate the usability and acceptability of the digital PCC tool and to explore age-related differences in user responses. Although digital health tools have been applied in areas such as contraception, pregnancy support, and menopausal symptoms, no prior studies have evaluated a PCC-focused digital tool within workplace health checkups in Japan. From an occupational health perspective, workplace health checkups may serve as a feasible platform for introducing reproductive health information to younger female employees.

## Materials and methods

Study design and setting

This cross-sectional pilot study was conducted between April and December 2023 in collaboration with multiple companies located in urban regions of Japan. The objective was to evaluate a digital PCC tool distributed during routine workplace health checkups. The survey was distributed through the companies’ health management offices, where female staff members received pamphlets containing a QR code linked to the PCC website.

Participants

The target population comprised female employees aged 18-40 years, a range considered to be within the reproductive age span. Eligible participants received a pamphlet containing a QR code linked to the digital PCC website. The QR code was provided either in person during workplace health checkups or via a follow-up email from the occupational health staff. An estimated total of 363 pamphlets were distributed, 340 accessed the tool, and 277 completed the questionnaire and were included in the final analysis. Participants were divided into three age groups (18-26, 27-34, and 35-40 years) because the limited sample size precluded the use of conventional five-year categories, and maternal age ≥35 years is generally recognized as advanced maternal age and is associated with increased pregnancy risk.

The age range of 18-40 years was selected because it aligns with the Adolescent and Young Adult (AYA) reproductive-age population, which is generally defined as individuals approximately 15-39 years of age [10,11]. Workplace health checkups in Japan are mandated under the Industrial Safety and Health Act and apply to employed adults aged 18 years and older [12,13]. The upper bound was extended to 40 years because this age represents a clinically relevant threshold within the reproductive period, particularly in the context of advanced maternal age (≥35 years), and remains consistent with the upper limit of the AYA framework.

Description of the digital tool

The digital PCC tool was designed for mobile accessibility and consisted of a brief, interactive questionnaire followed by personalized educational content. The content was generated based on the participants’ self-reported symptoms and concerns and covered several key reproductive health domains, including menstrual symptoms (such as dysmenorrhea and irregular cycles), awareness of gynecologic disorders (including uterine fibroids and endometriosis), knowledge of low-dose oral contraceptives and family planning options, understanding of appropriate body weight and body mass index, and engagement in preventive behaviors such as cervical and breast cancer screening.

Responses regarding menstrual symptoms, contraceptive use, body weight, and preventive behaviors were used to generate tailored messages. For example, women reporting irregular cycles received information on potential underlying factors and recommendations to seek medical consultation, whereas those who had not undergone cervical cancer screening received reminders to do so. The website used plain language and visually supportive elements to enhance comprehension for users across different levels of reproductive health literacy.

Data collection and outcome measures

Data were collected using a self-administered, anonymous questionnaire delivered through the digital tool. Participants completed the questionnaire after reviewing the educational content. Responses were collected electronically, and no personally identifiable information was obtained.

The questionnaire assessed perceived usefulness of the tool, ease of understanding, relevance to personal health, intention to seek medical consultation, and understanding of preconception care. Responses were analyzed overall and stratified by age group. The questionnaire items used to assess the five main outcome measures are provided in the Appendix.

Not all participants answered every questionnaire item. Therefore, the denominators for each outcome represent the number of respondents to that specific item (item-level response counts), and missing responses were treated as non-responses.

Ethical considerations

This study was conducted in accordance with the ethical principles outlined in the Declaration of Helsinki. Ethical approval was obtained from the Institutional Review Board (IRB) of Almeida Memorial Hospital (Approval No. 217). Participation was voluntary, and oral informed consent was obtained from all participants prior to participation. The IRB waived the requirement for written informed consent because the study involved an anonymous, minimal-risk questionnaire survey. All participants agreed to the use of their data for research purposes.

Statistical analysis

To examine differences in responses across the age groups, chi-square (χ²) tests were conducted for categorical variables related to each outcome item: perceived usefulness, ease of understanding, relevance to personal health, intention to seek medical consultation, and understanding of preconception care. All expected cell counts were ≥5; therefore, chi-square tests were appropriate for all analyses, and Fisher’s exact test was not required. A p-value of less than 0.05 was considered statistically significant. Because this study was exploratory in nature, no adjustments were made for multiple comparisons. Statistical analyses were performed using IBM SPSS Statistics, version 28.0 (IBM Corp., Armonk, NY).

## Results

A total of 277 participants completed at least one questionnaire item and were included in the analysis. Because not all participants answered every question, the number of respondents varied across outcomes. Table 1 summarizes the proportion of positive responses for each outcome by age group.

**Table 1:** Summary of Key Outcomes by Age Group Note: Values indicate the number of positive responses and the corresponding percentage within each age group. Because not all participants answered every questionnaire item, denominators vary across outcomes. Group differences were evaluated using chi-square (χ²) tests based on 3×2 contingency tables. All expected cell counts were ≥5; therefore, Fisher’s exact test was not required.

Outcome measure	18–26 years n/N (%)	27–34 years n/N (%)	35–40 years n/N (%)	Total n/N (%)	χ² value	p-value
Perceived usefulness	132/136 (97.1%)	87/102 (85.3%)	37/38 (97.4%)	256/276 (92.8%)	13.40	0.001
Ease of understanding	131/138 (94.9%)	93/101 (92.1%)	27/38 (71.1%)	251/277 (90.6%)	20.37	<0.001
Relevance to personal health	102/136 (75.0%)	79/101 (78.2%)	28/38 (73.7%)	209/275 (76.0%)	0.46	0.795
Intention to seek consultation	109/136 (80.1%)	64/84 (76.2%)	29/38 (76.3%)	202/258 (78.3%)	0.58	0.748
Understanding of PCC	120/127 (94.5%)	86/94 (91.5%)	34/35 (97.1%)	240/256 (93.8%)	1.63	0.444

Perceived usefulness was rated highly across all age groups (overall 256/276, 92.8%). A significant difference was observed between age groups (χ² = 13.40, p = 0.001), with the 27-34 years group reporting a lower proportion of positive responses compared with the younger and older groups.

Ease of understanding was also rated positively in most participants, although notable age-related variation was observed. The 35-40 years group reported a substantially lower proportion of easy-to-understand responses (27/38, 71.1%) compared with the 18-26 years (94.9%) and 27-34 years (92.1%) groups. This difference was statistically significant (χ² = 20.37, p < 0.001).

In contrast, relevance to personal health demonstrated no significant age-group differences (χ² = 0.46, p = 0.795), with similar proportions of participants across all age groups indicating that the information felt relevant to their health concerns.

Similarly, intention to seek medical consultation after reviewing the content showed no significant differences across age groups (χ² = 0.58, p = 0.748), with proportions of positive responses ranging from 76.2% to 80.1%.

Finally, understanding of PCC was high across all groups (overall 240/256, 93.8%), and no significant differences were detected between the age groups (χ² = 1.63, p = 0.444).

## Discussion

This pilot study evaluated the acceptability and perceived impact of a digital PCC tool delivered during workplace health checkups. The tool was generally well-received, with high levels of perceived usefulness and ease of understanding. Notably, significant age-group differences were observed in both perceived usefulness and ease of understanding, whereas no significant differences were found for relevance to personal health, intention to seek medical consultation, or understanding of PCC. These findings provide important insights into how digital PCC interventions may be received across different age groups in occupational settings.

Perceived usefulness was high overall but differed significantly by age group, with the 27-34 years group reporting a lower proportion of positive responses compared with younger and older participants. This pattern may reflect differing reproductive priorities among women in their late twenties and early thirties, who may be balancing career advancement with emerging family planning considerations. Prior research has suggested that perceptions of reproductive health needs can vary across life stages, potentially influencing the perceived utility of educational tools [1,2].

Ease of understanding also varied significantly by age group, with the 35-40 years group reporting lower ease-of-understanding ratings. This suggests that digital PCC tools may require tailoring for older users, who may differ from younger users in digital literacy levels or preferences for information presentation. Earlier studies on digital health materials have similarly shown that comprehension can differ by age, even when content is designed to be accessible [4].

In contrast, relevance to personal health and intention to seek medical consultation did not differ significantly by age. These findings indicate that once users engaged with the material, the perceived relevance of PCC concepts and their motivational impact were broadly consistent across age groups. The generally high intention to seek medical consultation suggests that digital PCC content may prompt users to reflect on their reproductive health needs. However, intention alone does not necessarily translate into behavioral change. Prior digital health research has shown that increases in awareness do not always lead to measurable changes in healthcare engagement [14,15]. Longitudinal follow-up studies will be important for determining whether digital PCC tools influence actual health-seeking behavior.

Understanding of PCC was high in all age groups, with no significant age-related differences. This suggests that the tool was effective in communicating core PCC concepts regardless of age, even though ease of understanding differed. From an occupational health perspective, this is encouraging: workplace health checkups may be a practical platform for delivering standardized PCC messaging to women of diverse ages, including those who may not routinely seek reproductive health care.

This study has several strengths, including its integration of PCC content into an established occupational health infrastructure and its use of a scalable digital platform. However, several limitations must be acknowledged. Not all participants answered every questionnaire item, leading to variable denominators and the possibility of response bias. In addition, the cross-sectional, exploratory design limits causal inference, and the relatively small number of participants in the oldest age group may have reduced the precision of age-stratified comparisons. Future studies with larger, more balanced samples and longitudinal designs are needed to clarify the impact of digital PCC interventions on actual health behaviors.

Overall, the findings suggest that digital PCC tools delivered during workplace health checkups are feasible and well accepted, with potential to enhance reproductive health awareness in working-age women. Tailoring content for different age groups and evaluating long-term outcomes will be important next steps in optimizing such interventions.

## Conclusions

This pilot study demonstrated that a digital PCC tool delivered during workplace health checkups was well accepted by women of reproductive age. Perceived usefulness and ease of understanding showed significant age-group differences, whereas relevance to personal health, intention to seek medical consultation, and understanding of PCC did not vary by age. These findings indicate that workplace settings can serve as a feasible platform for introducing digital PCC interventions, although tailoring content to different age groups-particularly older users-may improve usability. Because this exploratory study assessed self-reported perceptions rather than actual behaviors, future research with larger samples and longitudinal follow-up is needed to clarify how digital PCC tools influence real-world healthcare engagement and reproductive health outcomes.

## Appendices

Table 2 lists the questionnaire items used in the study. 

**Table 2:** Questionnaire Items Used in the Study Note: Responses were self-reported and collected anonymously via the digital tool. For analysis, positive responses were defined as “Yes” for binary items and as “Fully understand” or “Partially understand” for understanding of preconception care.

Domain	Questionnaire item	Response options
Perceived usefulness	Did you find the digital preconception care tool useful?	Yes / No
Ease of understanding	Was the content of the digital tool easy to understand?	Yes / No
Relevance to personal health	Did you find the content relevant to your personal health?	Yes / No
Intention to seek medical consultation	After using the tool, do you intend to seek consultation with a healthcare provider regarding preconception care?	Yes / No
Understanding of preconception care	After using the tool, do you feel that you understand preconception care?	Fully understand / Partially understand / Do not understand
